# The Sympathetic Ampulla

**DOI:** 10.5152/tjg.2020.18997

**Published:** 2023-01-01

**Authors:** James Gauci, Neville Azzopardi, Christine Cannataci, Kelvin Cortis, David Pisani, Snezana Bozanic

**Affiliations:** 1 Department of Gastroenterology, Mater Dei Hospital, Triq id-Donaturi tad-Demm, Msida, Malta; 2 Department of Radiology, Mater Dei Hospital, Triq id-Donaturi tad-Demm, Msida, Malta; 3 Department of Pathology, Mater Dei Hospital, Triq id-Donaturi tad-Demm, Msida, Malta

## CASE PRESENTATION

A 65-year-old lady was referred for cross-sectional imaging of the pancreas in view of a history of uncontrolled blood glucose. She was an ex-smoker and suffered from non-insulin-dependent diabetes mellitus and hypertension. She denied any abdominal symptoms, and the examination of her abdomen was unremarkable. Blood investigations were within normal limits.

**Figure 1.**
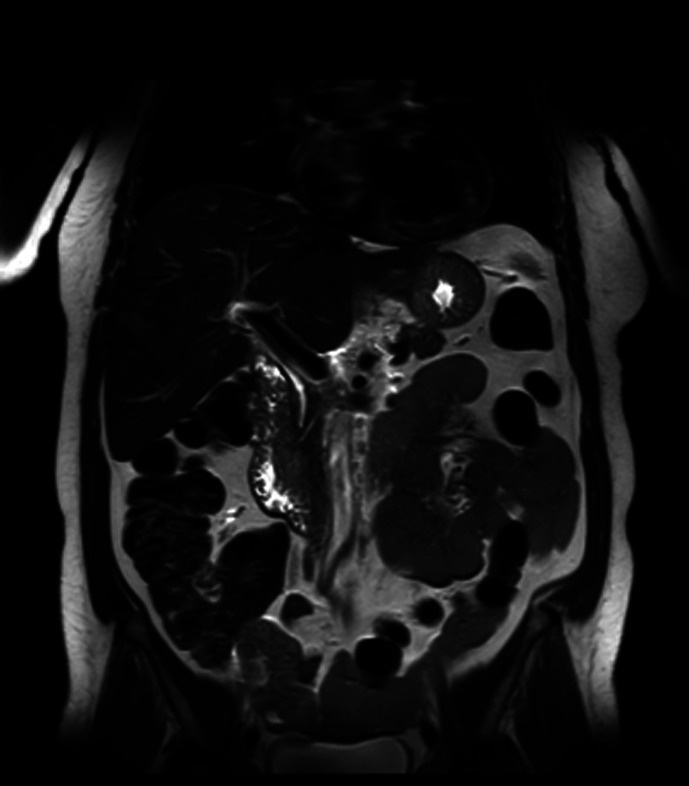
Coronal T2-weighted MR image showing a broad-based lesion at the ampulla. MR, magnetic resonance.

Magnetic resonance (MR) imaging incidentally revealed a broad-based ampullary lesion measuring 2 × 1.6 cm which demonstrated restricted diffusion and heterogeneous enhancement on dynamic contrast-enhanced sequences (Figure 1). Computed tomography (CT) confirmed the presence of a well-circumscribed mass that was isoattenuating when compared to the bowel wall. There was no evidence of extraduodenal invasion or vascular encasement.

**Figure 2.**
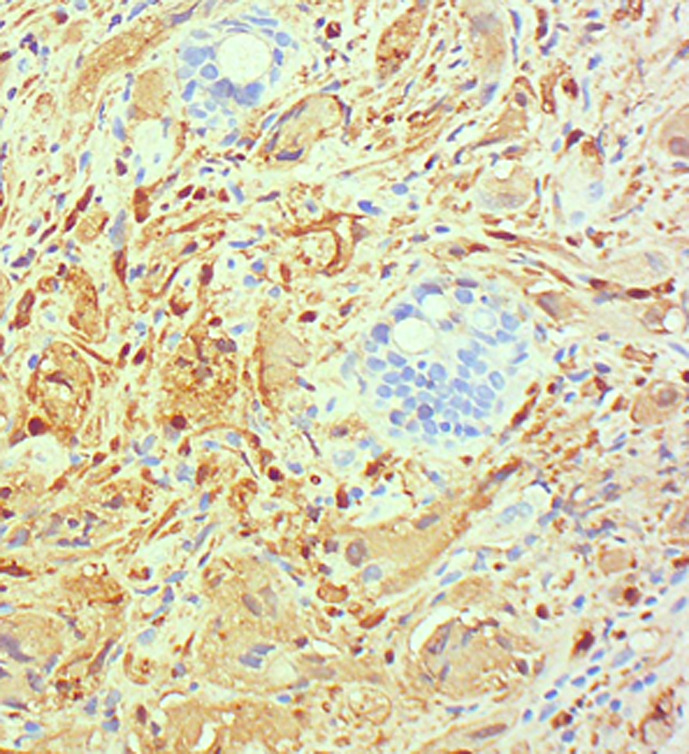
Review immunohistochemistry section showing S100-positive spindle cells.

A duodenoscopy established the presence of a prominent ampulla; multiple biopsies were taken through the bite-on-bite technique. Histological analysis revealed the proliferation of bland spindle-shaped mesenchymal cells with occasional ganglion cells. Both components were S-100 positive (Figure 2), whereas other strains (SMA, Desmin, CD117, DOG1) were negative. There were no mitotic figures or atypical cells. 

A panel of investigations to assess for endocrinological activity, including catecholamines and androgenic hormones, was normal. Following discussion at a multidisciplinary team meeting, a decision was taken to manage this lesion conservatively**. **Follow-up MR imaging will be organized yearly until any alteration in radiological features.

## DISCUSSION

The radiological and histological features of this incidental lesion are consistent with those of a ganglioneuroma, a rare neuronal tumor, in the ampulla. 

Ganglioneuromas are rare neuronal tumors derived from primordial neural crest cells, which are undifferentiated cells of the sympathetic nervous system.^1^ These lesions are composed of ganglion cells, Schwann cells, and fibrous tissue,^2^ in the absence of immature elements, thus distinguishing them from neuroblastomas and ganglioneuroblastomas.

These benign hamartomatous tumors can potentially arise anywhere along with the peripheral autonomic ganglion sites.^1^ They most commonly arise in the paravertebral sympathetic chains of the posterior mediastinum or paraspinal retroperitoneum, as well as in the adrenal medulla. They very rarely occur in the gastrointestinal tract, where they have a predilection for the colon and the duodenum. 

Patients may be asymptomatic or may present with non-specific symptoms such as abdominal pain.^1^ Lesions are usually endocrinologically inactive and are often discovered incidentally. Alternatively, they may secrete catecholamines, vasoactive intestinal polypeptide, or androgenic hormones, resulting in hypertension, diarrhea, and/or virilization.

On imaging, lesions are well-circumscribed and can be large.^3^ Less than a quarter will demonstrate fine and speckled calcifications. Computed tomography is the preferred imaging technique. It will characteristically reveal the presence of well-defined, solid, encapsulated masses that are iso- to hypoattenuating to muscle.^3^ It provides information regarding tumor size, organ of origin, tissue invasion, vascular encasement, adenopathy, and calcifications.^3^

Magnetic resonance imaging can allow for better tissue discrimination, hence enabling evaluation of the organ of origin and regional invasion.^3^ Lesions typically have relatively intermediate signal intensity on all sequences.^3^

The radiological appearance of these benign lesions is similar to that of malignant neuroblastomas and ganglioneuroblastomas. Differentiation is occasionally possible based on the lack of irregular contours, tissue invasion, and vessel encasing which are features of the more aggressive tumors.^1,3^ Moreover, calcifications tend to be discrete and punctate rather than coarse or amorphous.^3^ The finding of distant metastases also sets ganglioneuromas apart from their malignant counterparts.

Thorough tissue sampling is required in order to confirm the diagnosis.^1^ On gross pathologic analysis, ganglioneuromas are typically white, firm, well-circumscribed, and nodular tumors.^2^ Microscopically, the tumors are composed of an intimate admixture of spindle cells and ganglion cells. Immunohistochemistry of the spindle cells will reveal S-100-positivity, which confirms the tumor’s neural lineage.^2^ The presence of ganglion cells can be confirmed by staining for neuron-specific enolase and neurofilament; this will allow differentiation from other neurogenic spindle cell lesions such as neurofibromas.^2^

Complete surgical resection to confirm the diagnosis can be offered, however, this depends on the location. Endoscopic papillectomy is also a safe and effective option for ampullary lesions.^4^ Prognosis after resection is excellent and recurrence is rare.^1^ Malignant transformation has been very rarely reported,^5^ thus continued follow-up is required in patients who are managed conservatively.
